# Oxytocin Relieves Neuropathic Pain Through GABA Release and Presynaptic TRPV1 Inhibition in Spinal Cord

**DOI:** 10.3389/fnmol.2018.00248

**Published:** 2018-07-17

**Authors:** Wuping Sun, Qian Zhou, Xiyuan Ba, Xiaojin Feng, Xuexue Hu, Xiaoe Cheng, Tao Liu, Jing Guo, Lizu Xiao, Jin Jiang, Donglin Xiong, Yue Hao, Zixian Chen, Changyu Jiang

**Affiliations:** ^1^Department of Pain Medicine, Shenzhen Municipal Key Laboratory for Pain Medicine, Shenzhen Nanshan People’s Hospital and the 6th Affiliated Hospital, Health Science Center, Shenzhen University, Shenzhen, China; ^2^Department of Pain Medicine, The Third People’s Hospital of Hubei Province, Wuhan, China; ^3^Center for Experimental Medicine, The First Affiliated Hospital, Nanchang University, Nanchang, China; ^4^Department of Pharmacy, School of Medicine, Health Science Center, Shenzhen University, Shenzhen, China; ^5^Department of Orthopedic Surgery, Zhongshan Hospital, Fudan University, Shanghai, China

**Keywords:** oxytocin, neuropathic pain, TRPV1, GABA, spinal cord, mechanical allodynia, thermal hyperalgesia

## Abstract

**Objective:** Oxytocin (OT) is synthesized within the paraventricular nucleus and supraoptic nucleus of the hypothalamus. In addition to its role in uterine contraction, OT plays an important antinociceptive role; however, the underlying molecular mechanisms of antinociceptive role of OT remain elusive. We hypothesized that the antinociceptive effect of OT on neuropathic pain may occur via inhibition of TRPV1 activation in the spinal cord. The present study explores the antinociceptive role of OT and its mechanisms in neuropathic pain.

**Methods:** Partial sciatic nerve ligation (pSNL) was performed to induce neuropathic pain. Animal behaviors were measured using a set of electronic von Frey apparatus and hot plate. Electrophysiological recordings and molecular biological experiments were performed.

**Results:** Intrathecal administration of OT alleviated both mechanical allodynia and thermal hyperalgesia in pSNL rats (*n* = 6, per group, *P* < 0.0001, saline vs. OT group). Electrophysiological data revealed that OT significantly inhibited the enhancement of frequency and amplitude of spontaneous excitatory post-synaptic currents induced presynaptically by TRPV1 activation in the spinal cord. Moreover, the inhibitory effect of OT on capsaicin-induced facilitation of excitatory transmission was blocked by co-treatment with saclofen, while intrathecal administration of OT dramatically inhibited capsaicin-induced ongoing pain in rats, (*n* = 6, per group, *P* < 0.0001, saline vs. OT group). The paw withdrawal latency in response to heat stimulation was significantly impaired in TRPV1KO mice 3 days after pSNL upon OT (i.t.) treatment, compared with wild type mice (*n* = 6, *P* < 0.05). Finally, OT prevented TRPV1 up-regulation in spinal cords of pSNL model rats.

**Conclusion:** OT relieves neuropathic pain through GABA release and presynaptic TRPV1 inhibition in the spinal cord. OT and its receptor system might be an intriguing target for the treatment and prevention of neuropathic pain.

## Introduction

Neuropathic pain is a major public health concern due to its considerable impact on patients’ quality of life. Currently, clinical measures employed for neuropathic pain remain inadequate leading to multiple serious adverse effects and limited efficacy (Baron et al., 2010). Accordingly, safer and more effective analgesics are needed for neuropathic pain relief.

Oxytocin (OT), a nine-amino-acid peptide hormone and neuropeptide, is synthesized within the paraventricular nucleus (PVN) and supraoptic nucleus of the hypothalamus (Vandesande and Dierickx, 1975). OT is conveyed to the posterior pituitary gland and released into the bloodstream but fractional PVN neurons directly project to several other brain areas as well, including the spinal dorsal horn (Kiss and Mikkelsen, 2005). Moreover, neurons throughout the nervous system including those within the hippocampus, ventral medial hypothalamus, nucleus accumbens, amygdala, and spinal cord are known to express OT receptors (Reiter et al., 1994; Wrobel et al., 2011; Stoop, 2012). OT receptors are also expressed in the dorsal root ganglion (DRG) (Moreno-Lopez et al., 2013). There is growing evidence of the antinociceptive effects of OT in different pain models. For example in animal models, with neuropathic pain, intrathecal delivery of OT has been shown to be a significant analgesic (Miranda-Cardenas et al., 2006; Yang et al., 2007), while in animal models with inflammatory pain, OT release from parvocellular OT neurons suppresses nociception and promotes analgesia (Eliava et al., 2016). Systemic administration of OT produces analgesia in thermal, mechanical, and inflammatory stimulation (Schorscher-Petcu et al., 2010), and alleviates orofacial mechanical hypersensitivity associated with infraorbital nerve injury through vasopressin-1A receptors in rat trigeminal ganglia (Kubo et al., 2017). These studies provide promising evidence for antinociceptive action of OT. However, the mechanisms underlying these antinociceptive properties especially in neuropathic pain are still controversial, limiting their application in pain management. Understanding the analgesic mechanisms of OT may offer a potentially novel avenue for treating neuropathic pain.

Transient receptor potential channel vanilloid 1 (TRPV1), a non-selective cation channel, is a molecular integrator of painful stimuli such as noxious heat (>43°C) (Caterina et al., 1997; Caterina and Julius, 2001). TRPV1 is widely expressed throughout the nervous system, and has been shown to play a crucial role in nociceptive transmission under pathological forms of pain (Lappin et al., 2006; Spicarova and Palecek, 2008; Chen et al., 2009). Moreover, spinal synaptic plasticity accounts for the transition from acute to chronic pain in which TRPV1 plays a critical role pre- and post-synaptically (Choi et al., 2016). Various models of neuropathy have shown that TRPV1 expression in nociceptors is altered under pathological conditions (Biggs et al., 2007; Kim et al., 2008). OT has shown direct activation of TRPV1 in heterologous expression systems, planar lipid bilayer experiments, and isolated DRG neurons (Nersesyan et al., 2017). However, our previous study demonstrated that OT increases GABAergic inhibitory transmission in the superficial layers of the spinal cord via activation of OT receptors, which indicates GABA release upon OT application (Jiang et al., 2014). It has also been reported that GABA_B_ receptors form a complex with TRPV1 channel limiting the TRPV1-mediated thermal hyperalgesia, and that GABA as an endogenous GABA_B_ agonist modulates TRPV1 activity through GABA_B_ receptors (Hanack et al., 2015). Therefore, we hypothesize that OT exerts its antinociceptive effect on neuropathic pain by inhibiting activation of TRPV1 in the spinal cord.

In this study, detailed experiments were performed to investigate whether OT may inhibit spinal TRPV1 activation and expression. We found that OT relieved neuropathic pain through GABA release and presynaptic TRPV1 inhibition in the spinal cord, which further affected the behavior in neuropathic pain model rats.

## Materials and Methods

### Animals

**FIGURE 1:**
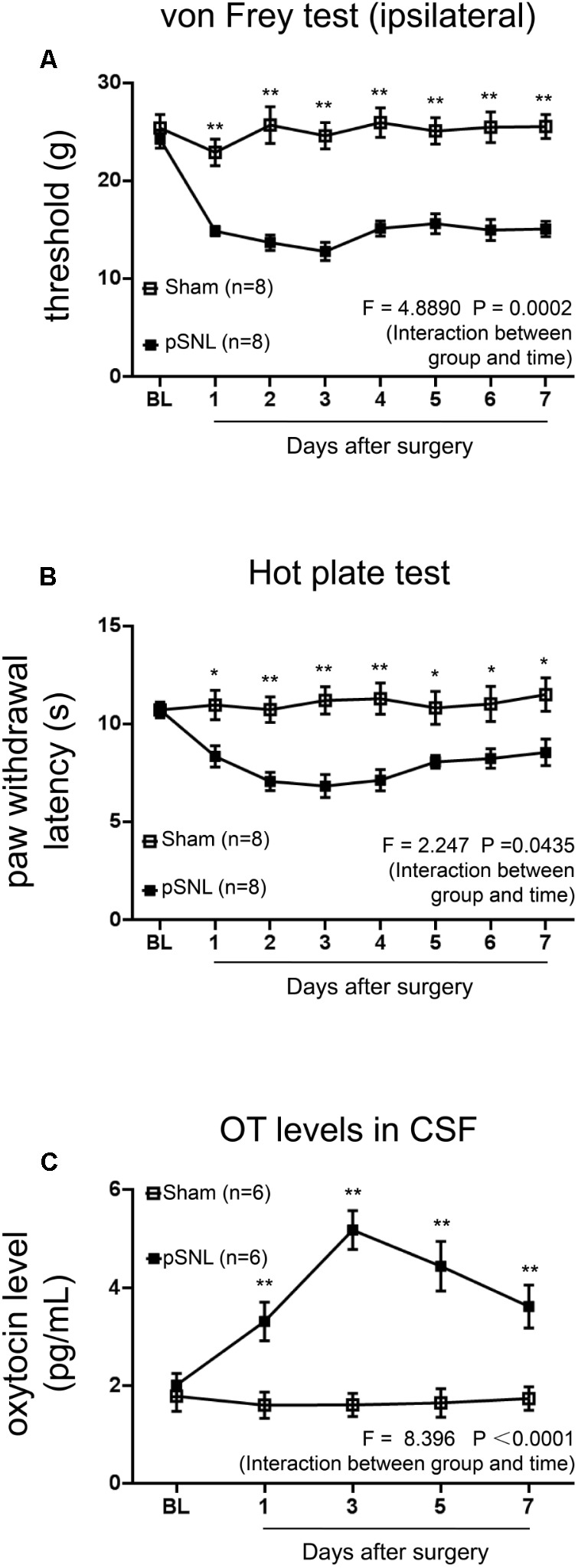
Behavioral tests and OT concentrations in CSF in rats after pSNL. **(A,B)** Mechanical **(A)** and thermal tests **(B)** in rats from sham and pSNL groups, *n* = 8. **(C)** OT concentrations in cerebrospinal fluid (CSF) of rats from sham and pSNL groups, *n* = 6. Mean ± SEM, ^∗^*P* < 0.05, ^∗∗^*P* < 0.01 vs. sham group at the same time point. Repeated measures ANOVA followed by Dunnett’s *post hoc* test were performed; statistical differences by Dunnett’s *post hoc* test were shown.

**FIGURE 2:**
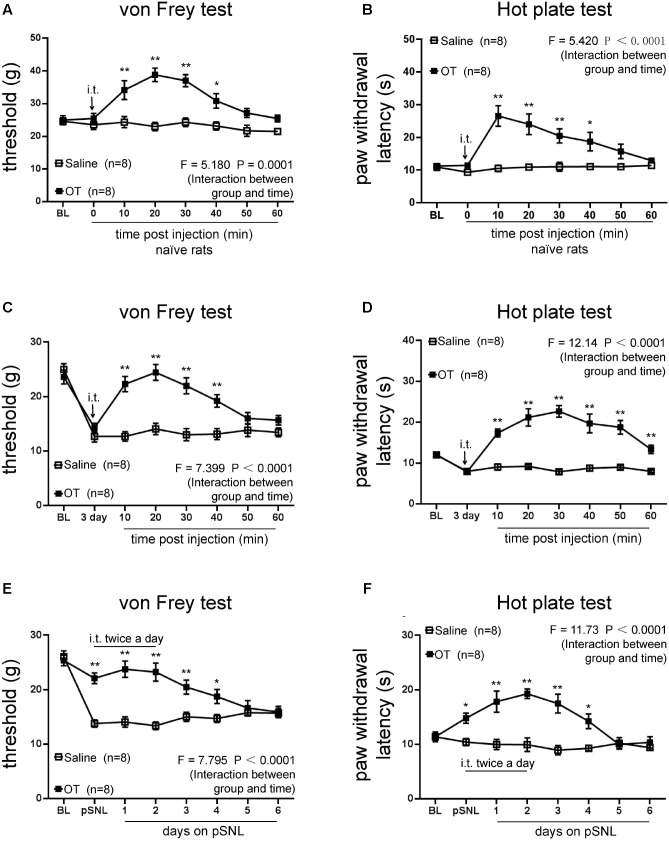
Effects of intrathecal injection of OT on rat behavioral tests. **(A,B)** Mechanical **(A)** and thermal tests **(B)** in naïve rats following one dose of OT administered intrathecally. **(C,D)** Mechanical tests **(C)** and thermal tests **(D)** in rats treated with or without intrathecal injection of OT at the third day on pSNL. **(E,F)** Mechanical tests **(E)** and thermal tests **(F)** in rats treated with or without continuous (twice per day for 3 days) intrathecal injection of OT. Mean ± SEM, *n* = 8; ^∗^*P* < 0.05, ^∗∗^*P* < 0.01 vs. pSNL group at the same time point. Repeated measures ANOVA followed by Dunnett’s *post hoc* test were performed; statistical differences by Dunnett’s *post hoc* test were shown.

**FIGURE 3:**
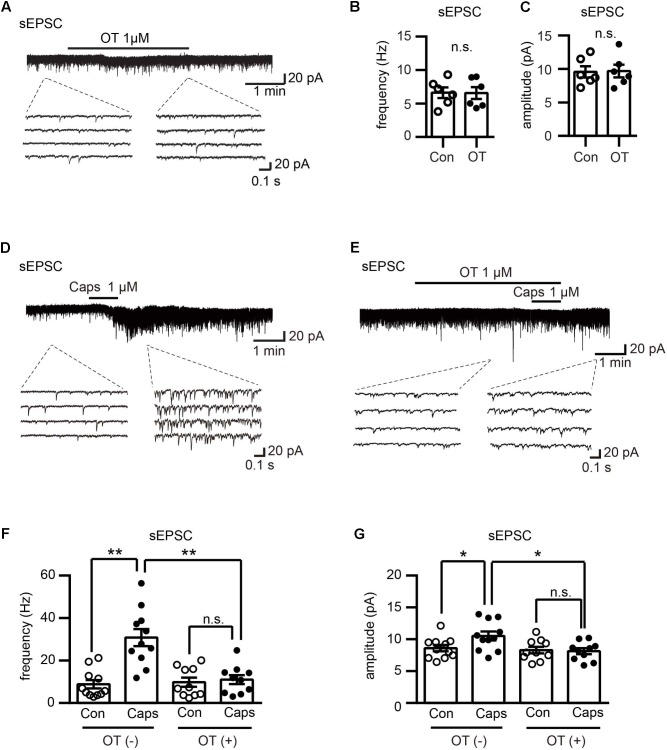
Effect of OT on capsaicin-induced facilitation of spontaneous excitatory transmission in Lamina II neurons. **(A)** Effect of OT (1 μM) on glutamatergic spontaneous excitatory transmission in Lamina II neurons. Lower panel is the emphasized current traces as the dash line indicated. **(B,C)** Analyzed data of the frequency **(B)** and amplitude **(C)** of spontaneous EPSC after OT application in Lamina II neurons. Mean ± SEM, *n* = 6. Paired Student’s *t*-test. **(D)** Effect of capsaicin (1 μM) on glutamatergic spontaneous excitatory transmission in Lamina II neurons. Lower panel is the emphasized current traces as the dash line indicated. **(E)** Effect of OT (1 μM) on capsaicin-induced glutamatergic spontaneous excitatory transmission in Lamina II neurons. Lower panel is the emphasized current traces as the dash line indicated. **(F,G)** Analysis of the frequency **(F)** and amplitude **(G)** of spontaneous EPSC after OT co-application with or without capsaicin in Lamina II neurons. Mean ± SEM, *n* = 11; ^∗^*P* < 0.05, ^∗∗^*P* < 0.01. One-way ANOVA followed by two-tailed *t*-test with Bonferroni correction.

**FIGURE 4:**
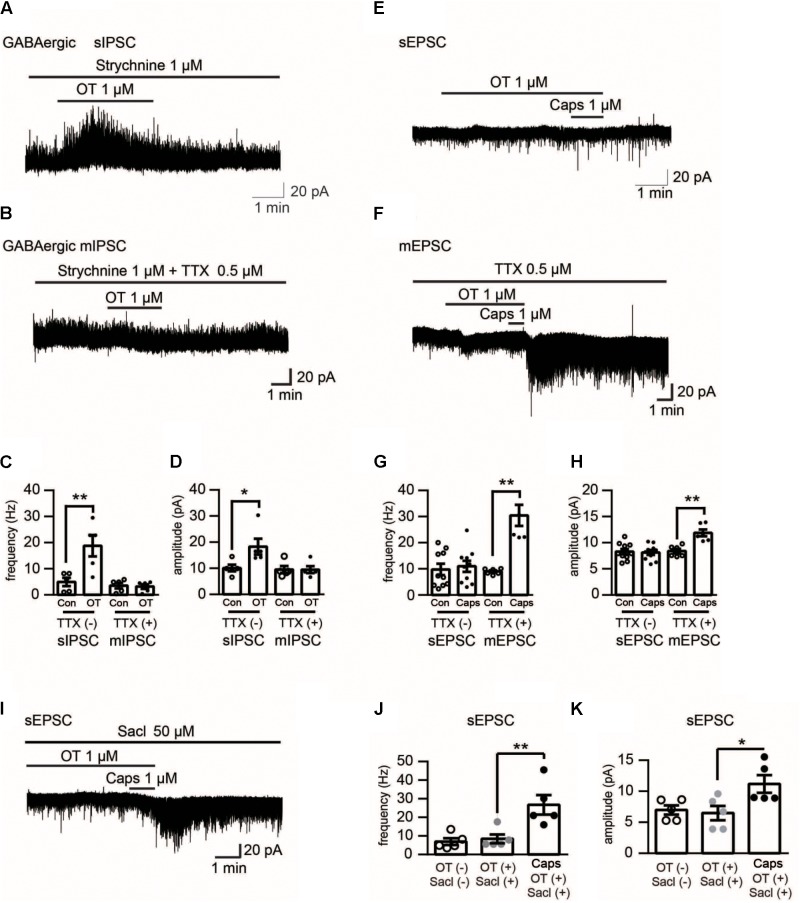
Electrophysiological recording in Lamina II neurons of rat spinal cord upon different pharmacological treatments. **(A)** Effect of OT (1 μM) on GABAergic spontaneous transmission in the presence of strychnine (1 μM), a glycine-receptor antagonist. **(B)** Effect of OT (1 μM) on mininature GABAergic transmission in the presence of strychnine (1 μM) and TTX (0.5 μM). **(C,D)** Analyzed data of the frequency **(C)** and amplitude **(D)** of spontaneous and mininature IPSC before and after OT application in Lamina II neurons. Mean ± SEM, *n* = 8. One-way ANOVA followed by two-tailed *t*-test with Bonferroni correction. **(E)** Effect of OT (1 μM) on capsaicin-induced glutamatergic spontaneous excitatory transmission in Lamina II neurons. **(F)** Effect of OT (1 μM) on capsaicin-induced mininature glutamatergic excitatory transmission in Lamina II neurons. **(G,H)** Analyzed data of the frequency **(G)** and amplitude **(H)** of capsaicin-induced spontaneous and mininature EPSC before and after OT application in Lamina II neurons. Mean ± SEM, *n* = 6. One-way ANOVA followed by two-tailed *t*-test with Bonferroni correction. **(I)** Effect of saclofen (50 μM) on the inhibitory effects of OT on capsaicin-induced glutamatergic spontaneous excitatory transmission in Lamina II neurons. **(J,K)** Analyzed data of the frequency **(G)** and amplitude **(H)** of capsaicin-induced spontaneous EPSC after saclofen co-application with or without OT in Lamina II neurons. Mean ± SEM, *n* = 6. One-way ANOVA followed by two-tailed *t*-test with Bonferroni correction.

**FIGURE 5:**
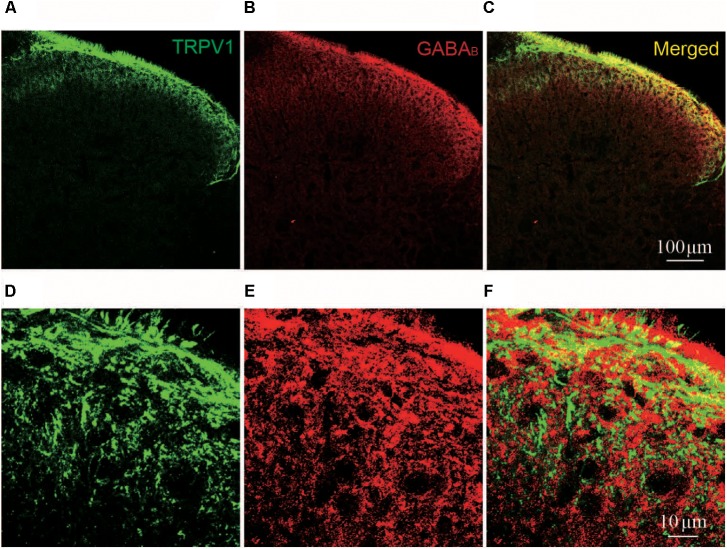
Immunofluorescent staining of TRPV1 and GABA_B_ in spinal cord. **(A–C)** Representative images of immunofluorescent staining of TRPV1 (green, **A**); GABA_B_ receptor (red, **B**); and merged TRPV1 and GABA_B_ receptor (yellow, **C**) in spinal cord of naïve rat. **(D–F)** Magnified images of immunofluorescent staining of TRPV1 (green, **D**); and GABA_B_ receptor (red, **E**); and merged TRPV1 and GABA_B_ receptor (yellow, **F**) in spinal cord of naïve rat.

**FIGURE 6:**
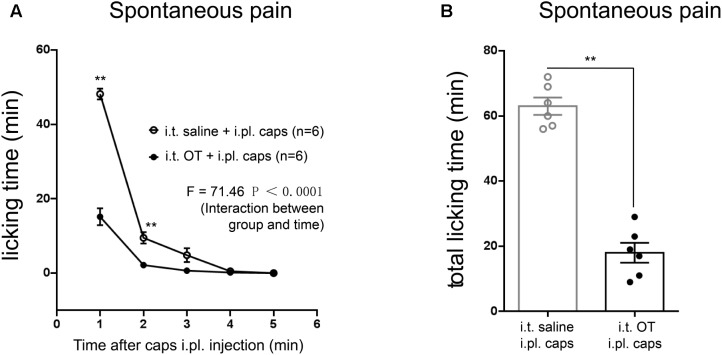
Effects of intrathecal injection of OT on capsaicin-induced licking behaviors in rats. **(A)** Time courses of capsaicin-induced licking time in rats treated with or without OT administered intrathecally. Mean ± SEM, *n* = 6; ^∗^*P* < 0.05, ^∗∗^*P* < 0.01 vs. saline group at the same time point. Repeated measures ANOVA followed by Dunnett’s *post hoc* test were performed; statistical differences by Dunnett’s *post hoc* test were shown. **(B)** Total licking time after capsaicin injection in rats treated with or without OT administered intrathecally. Mean ± SEM, *n* = 6; ^∗∗^*P* < 0.01 vs. saline group. Unpaired Student’s *t*-test.

**FIGURE 7:**
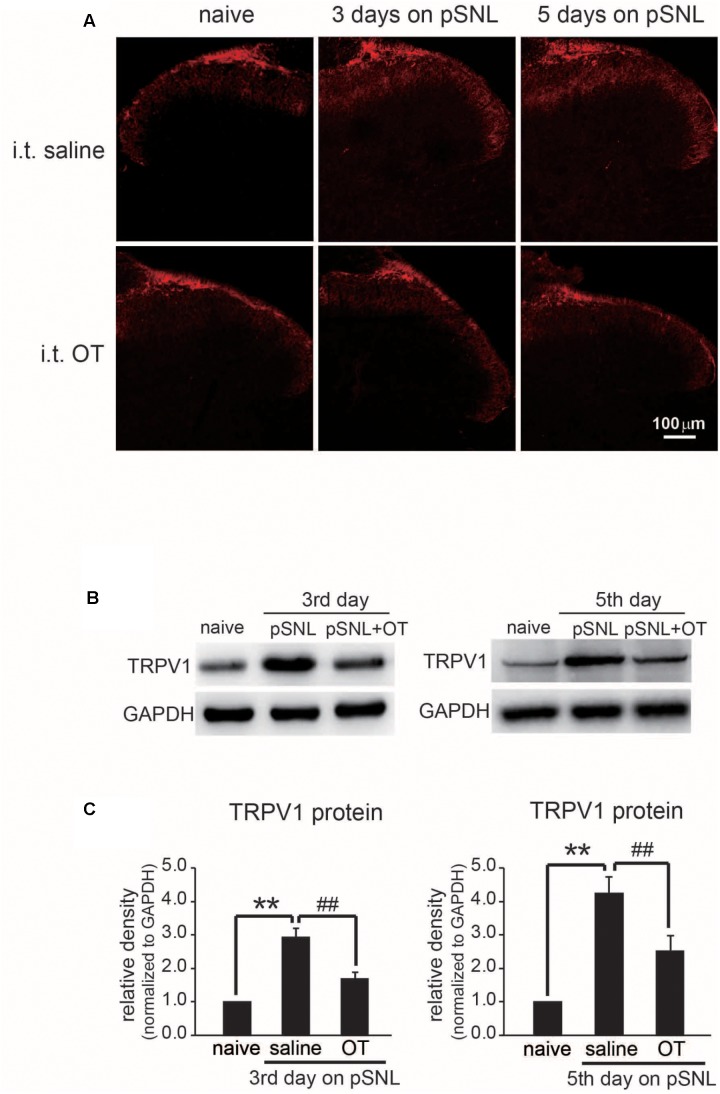
Effect of OT administration on the expression level of TRPV1 in rat spinal cords. **(A)** Representative images of immunohistochemistry staining of TRPV1 (red) in rat spinal cord after peripheral nerve injury treated intrathecally with saline or OT. **(B)** Western blot results of TRPV1 protein bands in rat spinal cord after peripheral nerve injury treated intrathecally with saline or OT. **(C)** Comparison of TRPV1 protein levels in rat spinal cord after peripheral nerve injury treated intrathecally with saline or OT. Mean ± SEM, *n* = 6; ^∗∗^*P* < 0.01 vs. pre group; ^##^*P* < 0.01 vs. pSNL group. One-way ANOVA followed by two-tailed *t*-test with Bonferroni correction.

**FIGURE 8:**
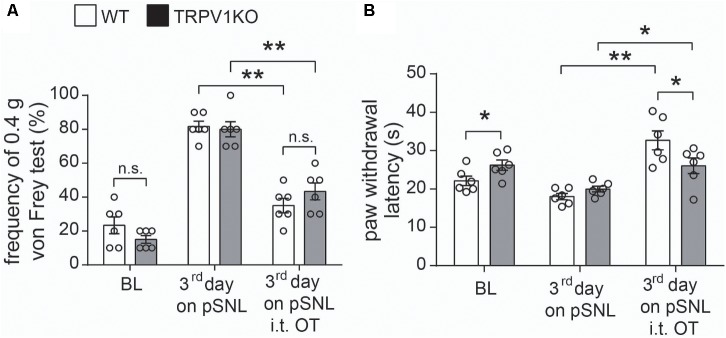
Effects of intrathecal injection of OT on WT and TRPV1KO mice after peripheral nerve injury. **(A)** Relative response frequency to von Frey test (0.4 g) of wild-type (WT) and TRPV1 knock-out (TRPV1KO) mice on the third day after pSNL treated with or without OT. **(B)** Paw withdrawal latency to thermal stimuli of WT and TRPV1KO mice on the third day after pSNL treated with or without OT. BL, baseline. Mean ± SEM, *n* = 6; ^∗^*P* < 0.05, ^∗∗^*P* < 0.01. One-way ANOVA followed by two-tailed *t*-test with Bonferroni correction.

All experimental procedures were carried out in accordance with the guidelines of the International Association for the Study of Pain and approved by the Institutional Animal Care and Use Committee at Shenzhen University. Male Sprague-Dawley rats (6–8 weeks of age) were used for most of the experiments (**Figures 1**–**7**). To explore the involvement of TRPV1 in the antinociceptive role of OT (**Figure 8**), male C57Bl/6J mice and TRPV1 knock-out (TRPV1KO) mice were purchased from Jackson Laboratory (Bar Harbor, ME, United States). The animals were housed with 2–3 rats/cage under a standard 12 h light/dark cycle at 23 ± 1°C, with free access to water and chow.

### Reagents

Oxytocin (catalog: H-2510) was purchased from Bachem AG (Switzerland). Tetrodotoxin (catalog: 1078) was obtained from Tocris (United States). Strychnine (catalog: S0532) and saclofen (catalog: S166) were purchased from Sigma-Aldrich (United States). Capsaicin (catalog: 034-11351) was purchased from Wako (Japan).

### Neuropathic Pain Model

The partial sciatic nerve ligation (pSNL) pain model was established according to previously described procedures (Seltzer et al., 1990). Briefly, animals were anesthetized with sodium pentobarbital (50 mg/kg, i.p.) and a tight ligation of approximately one-third to half the diameter of the right sciatic nerve (ipsilateral) was performed with 6–0 silk suture (Seltzer et al., 1990). In sham-operated rats, the nerve was exposed without ligation.

### Behavioral Tests

All behavioral tests were performed in a room with stable temperature and humidity. Rats and mice were habituated to the environment for at least 7 days before experiments.

To test mechanical sensitivity, rats and mice were placed on a metallic mesh floor covered with a plastic box, and a von Frey monofilament was applied from under the mesh floor to the plantar surface of the hind paw. Rat paw withdrawal responses to mechanical stimuli were measured using a set of electronic von Frey apparatuses (Ugo Basile, Italy). The weakest force (g) inducing withdrawal of the stimulated paw at least three times in five trials was referred to as the paw withdrawal threshold (Wang et al., 2016). Due to the fact that mice were not sensitive to the electronic von Frey apparatus, mechanical von Frey fibers (Stoelting, United States) were applied. The relative response frequency was measured by stimulating the hind-paw with a 0.4 g von Frey hair ten times (Chen et al., 2017).

To examine thermal hyperalgesia, a hot plate test (Ugo Basile, Italy) was applied. Each rat or mouse was placed on the plate; latency of paw withdrawal from the heat stimulus (52.5°C) was measured twice, separated by a 5-min interval. The average value was used as the latency of response (Chen et al., 2017).

To evaluate ongoing pain, the time that the rat spent licking, flinching and lifting its paw during a 5 min period following capsaicin application was recorded (Chen et al., 2017). All behavioral tests were performed in a blind fashion.

### Determination of OT Levels in CSF

Animals were deeply anesthetized using a small animal anesthesia machine (Matrx, VIP3000, United States) with 5% isoflurane diffused in the air and maintained with 2% isoflurane diffused in the air. Anesthetized rats were placed in a stereotaxic frame and the heads secured with ear bars. After removing the fur on the neck region, a needle was inserted horizontally and centrally into the cistern magna for cerebrospinal fluid (CSF) collection, without making any incision at this region. The volume of CSF collected was around 50 μL in each rat (Nirogi et al., 2009). OT levels in rat CSF were measured using an ELISA kit (K048-H1, Cayman Chemical, United States) according to instructions provided by the manufacturer. The absorbance of each plate was measured using a Bio-Rad model 550 microplate reader (Bio-Rad, United States). Within- and between-assay precision values for replicate quality control samples were within 4.3% and 5.9%, respectively.

### Intrathecal Drug Administration

Intrathecal catheterization was performed on intact rats or mice 7 days before pSNL surgery. Animals were deeply anesthetized using a small animal anesthesia machine (Matrx, VIP3000, United States) with 5% isoflurane diffused in the air and maintained with 2% isoflurane diffused in the air. A polyethylene catheter (PE-10 polyethylene catheter was used for rats, while an artificially stretched PE-10 polyethylene catheter was used for mice) filled with saline was inserted from the atlanto-occipital membrane into the subarachnoid space. The catheter was then passed to reach the lumbar enlargement of the spinal cord. Another tip of catheter was tightly tied and implanted subcutaneously until OT injection. For experiments with rats, OT (0.1 μg in 10 μL, i.t.) or saline was administrated either once at the third day on pSNL, or twice per day for a continuous 3 days after pSNL, as previously reported (Yu et al., 2003; Yang et al., 2007). For experiments with mice, the same dosage of either OT (0.1 μg in 10 μL, i.t.) or saline was injected. Only rats and mice showing no motor apparent dysfunction were used for experiments.

### Intraplantar Injection

To explore the involvement of TRPV1 in the antinociceptive role of OT, local intraplantar (i.pl.) injection of capsaicin was performed on naïve rats without anesthesia. Capsaicin (10 μg in 10 μL) or saline was injected using a Hamilton microsyringe with a 30-G needle.

### Spinal Cord Slice Preparation

Adult (6–8 weeks old; 200–300 g) male Sprague-Dawley rats were anesthetized with sodium pentobarbital (50 mg/kg, i.p.). The lumbosacral spinal cord was carefully removed and quickly placed in ice-cold Krebs solution (in mM: NaCl 117, KCl 3.6, MgCl_2_ 1.2, CaCl_2_ 2.5, NaHCO_3_ 25, NaH_2_PO_4_ 1.2 and glucose 11) saturated with 95% O_2_ and 5% CO_2_ as previously described (Peng et al., 2017). Transverse slices (300–400 μm) were prepared using a vibrating micro slicer (Leica, United States), and incubated at 32°C for at least 30 min in Krebs equilibrated with 95% O_2_ and 5% CO_2_.

### Electrophysiological Recording

Each slice was placed in the recording chamber and completely submerged and superfused at a rate of 2–4 ml/min with Krebs which was saturated with 95% O_2_ and 5% CO_2_ and maintained at room temperature. Lamina II neurons in lumbar segments were identified as a translucent band under a microscope (Olympus, Japan) with light transmitted from below, as previously described (Jiang et al., 2009). Whole-cell voltage-clamp recordings were made from lamina II neurons using patch-pipettes fabricated from thin-walled, fiber-filled capillaries. Patch-pipette solution contained (in mM): K-gluconate 135, KCl 5, CaCl_2_ 0.5, MgCl_2_ 2, EGTA 5, HEPES 5, Mg-ATP 5; or Cs_2_SO_4_ 110, CaCl_2_ 0.5, MgCl_2_ 2, EGTA 5, HEPES 5, Mg-ATP 5, tetraethylammonium (TEA)-Cl 5 (pH = 7.2) (Jiang et al., 2014).

The former and latter solutions were used to record excitatory and inhibitory post-synaptic currents (EPSCs and IPSCs), respectively. EPSC recordings were made at a holding potential (V_H_) of -70 mV, where no IPSCs were observed, since the reversal potential for IPSCs was near -70 mV. IPSCs were recorded at a V_H_ of 0 mV, where EPSCs were invisible as reversal potential for EPSCs was close to 0 mV. Cs^+^ and TEA were used to block K^+^ channels expressed in the recorded neurons, and thus to easily shift V_H_ from -70 mV to 0 mV. GABAergic IPSCs were obtained in the presence of a glycine-receptor antagonist, strychnine (1 μM). Signals were acquired using an Axopatch 200B amplifier (Molecular Devices, United States). The currents obtained in the voltage clamp mode were low-pass-filtered at 3 kHz and digitized at 333 kHz with an A/D converter (Molecular Devices, United States). The data were stored and analyzed with a personal computer using pCLAMP 9.2 software. EPSC and IPSC events were detected and analyzed using Mini Analysis Program ver. 6.0.

### Immunofluorescence

Lumbar (L) 4, L5, and L6 segmental spinal cord sample sections (10 μm) were cut in a cryostat (Leica, Germany), mounted on gelatin-coated glass slides, and processed for immunofluorescent staining. Sections were blocked and then incubated overnight at 4°C with TRPV1 antibody (Santa Cruz, CA, United States) and GABA_B_ antibody (Millipore, Germany). Sections were then incubated for 30 min at 25°C with AF488- and AF647-conjugated secondary antibodies (Jackson Immuno-Research, United States). Fluorescence images were captured using a FV1000 confocal microscope (Olympus, Japan).

### Western Blotting

Upon completion of behavioral testing, rats were anesthetized with 10% chloral hydrate; L4–L6 spinal cords were removed and frozen in liquid nitrogen. Samples were lysed in 100 μL RIPA lysis buffer with complete protease inhibitor cocktail (Roche Molecular Biochemicals, Switzerland). The supernatants were collected after centrifugation. The samples were denaturized at 95°C for 5 min, separated on an 8% SDS-PAGE gel, and transferred onto a PVDF membrane. The membrane was blocked using BSA reagent (Sigma, United States) at 4°C overnight. After three washes with PBS-T (0.1% Triton X-100), the membrane was incubated at room temperature for 1 h with a rabbit anti-TRPV1 (sc-28759, Santa Cruz, CA, United States), then incubated with a goat anti-rabbit IgG HRP-linked antibody (ab6721, Abcam, United Kingdom). Signals were visualized using an ECL kit (32106, Pierce, United States), and the PVDF membrane was photographed using a ChemiDoc^TM^ XRS+ imaging system (Bio-Rad, United States). The densities of protein blots were analyzed by using ImageJ software (National Institutes of Health, United States).

### Statistical Analysis

All statistical analyses were performed using SPSS 18.0 software (SPSS, Inc., Chicago, IL, United States). To calculate the appropriate sample size for behavioral tests, a power analysis was conducted on the basis of our preliminary study. The mean paw mechanical threshold (primary outcome) at baseline was 26.00 g, while the data (third day post pSNL surgery) in sham, pSNL + OT (i.t.) and pSNL + saline (i.t.) groups were 24.27 ± 0.96 g, 23.56 ± 1.98 g, and 15.25 ± 0.70 g, respectively. Thus, when compared with the control group, we expected a mean difference of 8.31 g among treatment groups.

An *a priori* algorithm was utilized to find the necessary sample size for analysis of variance (ANOVA) with repeated measures. Therefore, a sample size of six rats/group was required to identify a statistical difference (α = 5%) with a power (β-value) of 0.8. We considered the increase of the sample size to 6–8 rats/group for acquiring accurate and reasonable results. Group data are presented as the mean ± SEM. Statistical differences in the data were evaluated by unpaired Student’s *t*-tests, one-way ANOVA followed by multiple *t*-tests with Bonferroni correction or repeated measures ANOVA followed by Dunnett’s *post hoc* tests as appropriate. Results were considered significant at *P* < 0.05.

## Results

### OT Concentrations in CSF Were Significantly Increased in Rats After pSNL

First, we performed mechanical tests and thermal tests in rats after pSNL surgery. Mechanical threshold (**Figure 1A**; *n* = 8, per group; *F* = 4.8890, *P* = 0.0002, repeated ANOVA) and thermal paw withdrawal latency (**Figure 1B**; *n* = 8, per group; *F* = 2.247, *P* = 0.0435, repeated ANOVA) were significantly reduced in pSNL group rats from the first day after ligation, compared to sham group rats, and sustained for at least 7 days. OT concentrations in CSF were significantly increased in rats from the first day after pSNL (**Figure 1C**; *n* = 6 per group; *F* = 8.396, *P* < 0.0001, repeated ANOVA), peaking on the third day after pSNL from the initial 2.01 ± 0.24 to 5.18 ± 0.40 (pg/mL), indicating a physiological increase of OT levels in CSF upon peripheral nerve injury.

### Intrathecal Injection of OT Significantly Increased the Threshold of Behavioral Tests and Alleviated Neuropathic Pain

To explore the antinociceptive effects of OT on neuropathic pain, we analyzed the rat behavior after intrathecal administration of OT. Mechanical thresholds (**Figure 2A**; *n* = 8 per group; *F* = 5.180, *P* = 0.0001, repeated ANOVA) and thermal paw withdrawal latencies (**Figure 2B**; *n* = 8 per group; *F* = 5.420, *P* < 0.0011, repeated ANOVA) upon stimulation were significantly increased after intrathecal OT injection in naïve rats, compared to the saline injection group. We then tested the effects of OT on pSNL rats. One dose of intrathecally injected OT significantly increased mechanical thresholds (**Figure 2C**; *n* = 8 per group; *F* = 7.399, *P* < 0.0001, repeated ANOVA) and paw withdrawal latencies upon thermal stimulation (**Figure 2D**; *n* = 8 per group; *F* = 12.14, *P* < 0.0001, repeated ANOVA) on the rats 3 days after pSNL, and the antinociceptive effects of OT were consistent within an hour.

In pSNL rats injected intrathecally with OT twice a day for 3 days, mechanical thresholds (**Figure 2E**; *n* = 8 per group; *F* = 7.795, *P* < 0.0001, repeated ANOVA) and paw withdrawal latencies upon thermal stimulation (**Figure 2F**; *n* = 8 per group; *F* = 11.73, *P* < 0.0001, repeated ANOVA) were dramatically increased, and lasted 2 days after OT injection. Our data revealed that not only acute but also continuous intrathecal injection of OT increased mechanical thresholds and paw withdrawal latencies (**Figures 2C–F**).

### OT Inhibited Capsaicin-Induced Facilitation of Spontaneous Excitatory Transmission in Lamina II Neurons

To explore the mechanism of the antinociceptive effects of OT on neuropathic pain rats, we performed electrophysiological recordings by using slices from rat spinal cord. We found that OT (1 μM) perfusion for 3 min did not affect the frequency and amplitude of spontaneous EPSCs in all examined Lamina II neurons (*n* = 6, *P* = 0.9 and *P* = 0.716, respectively, see the lower traces of **Figures 3A–C**), from 6.62 ± 0.80 Hz to 6.57 ± 0.88 Hz, and 9.57 ± 0.84 pA to 9.68 ± 0.95 pA, respectively. These results are consistent with our previous work (Jiang et al., 2014). On the other hand, capsaicin (1 μM, a specific TRPV1 agonist) perfusion for 1 min, the spontaneous EPSCs frequency increased gradually over time and peaked at around 2 min after capsaicin addition (**Figures 3D,F**; *n* = 11, *P* < 0.0001, one-way ANOVA), from 8.81 ± 1.96 Hz to 30.78 ± 4.00 Hz. Besides, this enhancement was accompanied by an increase in spontaneous EPSC amplitude (**Figures 3D,G**, *n* = 11, *P* = 0.0016, one-way ANOVA), 8.60 ± 0.49 pA to 10.52 ± 0.69 pA.

Next, we found that the effects of capsaicin were inhibited in the presence of OT (1 μM) in both frequency and amplitude of spontaneous EPSCs, from 9.77 ± 2.16 Hz to 10.97 ± 2.12 Hz, and 8.30 ± 0.51 pA to 8.13 ± 0.46 pA, respectively (**Figures 3E–G**; *n* = 10, *P* = 0.3774 and *P* = 0.5172, respectively).

### GABA_B_ Receptor Mediated the Effects of OT on Capsaicin-Induced Facilitation of Spontaneous Excitatory Transmission in Lamina II Neurons

To further clarify the effect of OT on capsaicin-induced facilitation of spontaneous excitatory transmission, we tested the effects of OT on spontaneous and miniature GABA release. Firstly, we found that OT (1 μM) increased the frequency and amplitude of spontaneous GABAergic IPSCs in the presence of strychnine (1 μM), a glycine-receptor antagonist, from 4.94 ± 1.59 Hz to 18.80 ± 4.10 Hz, and 10.01 ± 1.27 pA to 18.30 ± 3.05 pA, respectively (**Figures 4A,C,D**; *n* = 5, *P* = 0.009 and *P* = 0.0138, respectively, one-way ANOVA). However, as seen from **Figures 4B–D** (*n* = 5, *P* = 0.117 and *P* = 0.7519, respectively, one-way ANOVA), OT did not alter the frequency and amplitude of miniature GABAergic IPSCs in the presence of strychnine (1 μM) and tetrodotoxin (TTX) (0.5 μM), a voltage gated Na^+^-channel blocker. On the other hand, the blockage effect of OT (1 μM) on capsaicin-induced excitatory facilitation was disappeared in the presence of TTX. In the presence of OT and TTX, capsaicin (1 μM) increased the frequency and amplitude of miniature EPSCs by 249.82 ± 57.31% and 52.74 ± 11.30 % (**Figures 4E–H**; *n* = 6; *P* = 0.0043 and *P* = 0.0025, respectively, one-way ANOVA). Finally, we checked the effect of saclofen, a GABA_B_ receptor antagonist. Our data indicated that in the presence of saclofen (50 μM) and OT (1 μM), capsaicin increased the frequency and amplitude of spontaneous EPSCs (**Figures 4I–K**; *n* = 6; *P* = 0.0043 and *P* = 0.028, respectively, one-way ANOVA). The frequency and amplitude of spontaneous of EPSCs were increased from 7.67 ± 2.32 Hz to 27.68 ± 5.47 Hz, and 7.00 ± 0.75 pA to 9.80 ± 1.42 pA, respectively. In this study, spontaneous excitatory transmission was not affected by TTX or saclofen itself.

### TRPV1 and GABA_B_ Receptor Were Co-expressed in Spinal Cord Lamina II

OT inhibits TRPV1 activation through GABA-GABA_B_ receptor. Therefore, we further asked whether TRPV1 co-expressed with GABA_B_ receptor in spinal cord superficial layers. We examined the colocalization of TRPV1 and GABA_B_ receptor in slice of spinal cord from naïve rats using immunohistochemistry method. We found that TRPV1 (Green) and GABA_B_ receptor (Red) signals in superficial layers of spinal cord (**Figures 5A,B,D,E**). And their colocalization was also observed when merged TRPV1 and GABA_B_ receptor staining images (**Figures 5C,F**).

### Intrathecal Injection of OT Significantly Reduced TRPV1-Mediated Licking Behavioral in Rats

In order to address whether TRPV1 activation is involved in OT analgesia, we next examined the effect of OT on capsaicin-induced licking behaviors in rats. Intraplantar (i.pl.) injection of capsaicin (10 μg/μL) induced typical licking behavior, compared to saline (i.pl.) group (*n* = 6 per group; *F* = 125.0, *P* < 0.0001, repeated ANOVA; data not shown). However, 10 min pre-treatment of OT (i.t.) significantly reduced the licking induced by capsaicin injection (**Figure 6A**; *n* = 6 per group; *F* = 73.51, *P* < 0.0001, repeated ANOVA). And the total licking time was also dramatically decreased in OT-treated group, compared to saline-treated group (**Figure 6A**; *n* = 6 per group; *P* < 0.0001, un-paired *t*-test).

### OT Administration Also Suppressed TRPV1 Expression Level Up-Regulation in Spinal Cord Superficial Layers of pSNL Rats

Our previous data demonstrated that TRPV1 is involved in antinociceptive effect of OT. It has also been reported that TRPV1 expression level was up-regulated in spinal cord superficial layers of neuropathic pain rats (Kanai et al., 2005). We therefore checked whether OT regulates TRPV1 expression in spinal cord of pSNL rats. We checked the expression levels of TRPV1 in different stages of neuropathic pain by either immunohistochemistry staining or western blot methods. Our immunostaining data revealed that TRPV1 protein expression was dramatically increased in spinal cord of rats upon pSNL surgery, and the up-regulation of TRPV1 expression in spinal cord of rats was significantly suppressed by OT administration (**Figure 7A**). We further confirmed these results by western blot method. Western blot data also demonstrated that TRPV1 protein levels were dramatically increased in spinal cord of rats either at 3 days or 5 days on pSNL, but these increases in TRPV1 expression were significantly blocked upon OT injection intrathecally (**Figures 7B,C**; *n* = 6, *P* < 0.01, one-way ANOVA).

### Antinociceptive Effect of OT on Thermal Hyperalgesia in pSNL Model May Partially Act Through TRPV1 Activation in the Spinal Cord of Mice

To confirm the involvement of TRPV1 in the antinociceptive role of OT, we tested the antinociceptive effect of OT in TRPV1KO mice. As previously reported (Caterina et al., 2000), the basal paw withdrawal latency to thermal stimulation was significantly longer in TRPV1KO mice than in wild-type (WT) mice. Our results showed that the animals treated with OT (i.t.) significantly recovered the response frequency to mechanical stimulation (0.4 g) and the paw withdrawal latency by heat stimulation not only in WT but also in TRPV1KO mice 3 days after pSNL (**Figures 8A,B**, *n* = 6, per group, *P* < 0.05, *P* < 0.01, one-way ANOVA). Statistical analysis results showed that there was no significant difference in the response frequency to mechanical stimulation (**Figure 8A**, *n* = 6, per group, *P* = 0.41, one-way ANOVA) between WT and TRPV1KO mice 3 days after pSNL upon OT (i.t.) treatment, although the response frequency was slightly higher in TRPV1KO mice 3 days after pSNL. However, the paw withdrawal latency in response to heat stimulation was significantly impaired in TRPV1KO mice 3 days after pSNL upon OT (i.t.) treatment (**Figure 8B**, *n* = 6, per group, *P* < 0.05, one-way ANOVA).

## Discussion

Oxytocin is a nine-amino acid peptide hormone and neuropeptide, which promotes uterine contractions. Stimulation of the anterior part of the PVN has been reported to increase OT concentration, producing antinociceptive effects in animals with sciatic nerve ligation (Martinez-Lorenzana et al., 2008). A previous study reported that OT inhibited glutamate-induced neuronal firing in the spinal dorsal horn (Condes-Lara et al., 2003). OT-induced antinociception in the spinal cord is mediated by a subpopulation of glutamatergic neurons in lamina I–II which amplify GABAergic inhibition (Breton et al., 2008). Moreover, hypothalamic PVN stimulation-induced oxytocinergic inhibition in dorsal horn neurons was mediated by GABA (Rojas-Piloni et al., 2007). Here, we demonstrated that OT relieves neuropathic pain through GABA release and presynaptic TRPV1 inhibition in the spinal cord; thus, OT may be a novel endogenous analgesic mechanism through GABA release and TRPV1 inhibition.

We found that OT levels in CSF increased significantly 1 day after pSNL surgery and peaking 3 days post-surgery (**Figure 1C**), suggesting that OT might play a role in neuropathic pain. Although a potential bias using ELISA kit to measure OT concentration was reported between the extracted and unextracted samples (Szeto et al., 2011), we followed sample extraction steps when examining OT levels in samples. OT levels were consistently elevated in the spinal cords of rats exhibiting pain symptoms (Juif et al., 2013). Gutierrez et al. (2013) reported peripheral nerve injury-induced hypersensitivity was reversed in postpartum period rats, while this phenomenon was not observed in rats after delivery and separation of their pups. Other studies have suggested that OT could be part of endogenous antinociceptive system (Condes-Lara et al., 2009; Eliava et al., 2016). Moreover, in male rats, a group of oxytocinergic neurons have been identified within the hypothalamic PVN which respond to noxious stimulation projecting to the spine and modulating nociceptive transmission (Condes-Lara et al., 2009).

Behavioral studies have shown that intrathecal administration of OT significantly ameliorates either mechanical or thermal pain behavior in neuropathic pain model rats. It should be noted that one intrathecal injection of OT alone provides pain relief for an hour. On the other hand, OT was administered intrathecally at the first 3 days continuously after peripheral nerve injury, resulting in the prevention of neuropathic pain lasting 2 days after OT administration (**Figure 2**). This data suggests multiple intrathecal applications of OT may prevent the development of neuropathic pain upon peripheral nerve injury. Condes-Lara et al. (2009) also noted the analgesic effects of OT in neuropathic rats, while Reeta et al. (2006). found OT to display an important antinociceptive effect on a formalin-induced tonic continuous pain response in mice.

Electrophysiological study found OT inhibited TRPV1 activation in the superficial dorsal horn of the spinal cord (**Figure 3**). TRPV1 is known to play a crucial role in nociceptive transmission during pathological forms of pain (Lappin et al., 2006; Spicarova and Palecek, 2008; Chen et al., 2009). In peripheral mechanisms of acute and chronic pain, TRPV1 activation and up-regulation induced nociceptor sensitization (Ji et al., 2002; Cheng and Ji, 2008). TRPV1 is also involved in synaptic plasticity, which accounts for the progression of acute to chronic pain (Choi et al., 2016). Consistent with our previous study (Jiang et al., 2009), capsaicin increased the frequency of spontaneous EPSCs with minimal change in amplitude. Spontaneous EPSCs are irritated by a spontaneous release of glutamate from presynaptic vesicle, and this spontaneous release is in calcium dependent manner (Rusakov, 2006). TRPV1 channel has a higher Ca^2+^ permeability and the activation of TRPV1 induces Ca^2+^ influx resulting depolarization which in turn activates voltage-gated Ca^2+^ channels (Caterina et al., 1997; Marshall et al., 2003). Therefore, the frequency increase would be due to the facts that capsaicin activated presynaptic TRPV1 channel leading to an increase of spontaneous EPSCs.

On the other hand, we did not observe changes in the spontaneous release of presynaptic excitatory neurotransmitters in response to OT. However, some of the recorded neurons depolarized upon OT treatment, and this depolarization was mediated by OT receptors, but not vasopressin-V1a receptors, and was resistant to AMPA receptor antagonists, Ca^2+^-free Krebs and TTX (Jiang et al., 2014). This data suggests OT receptors are functionally expressed post-synaptically, but not pre-synaptically. In agreement with our finding, Robinson et al. (2002) found OT inhibited nociceptive transmission in spinal cord by post-synaptic OT receptor activation. Moreno-Lopez et al. (2013) have also reported that OT receptors are not expressed in the central ending terminals of nociceptive fibers, though they are expressed predominantly in non-peptidergic C-fiber cell bodies in the DRG.

Based on our data, we conclude that OT-induced inhibition of TRPV1 activation in the spinal cord occurs indirectly. OT has been found to directly activate TRPV1 demonstrated in heterologous expression systems, planar lipid bilayer experiments, and isolated DRG neurons (Nersesyan et al., 2017). However, in our previous and current study, we did not observe OT (0.01–5 μM) altered excitatory transmission in spinal cords (Jiang et al., 2014). Moreover, they reported that OT-elicited calcium influx is mediated by TRPV1 channels in native and heterologous expression systems; we found that OT-induced calcium response in superficial dorsal horn neurons is mediated by intracellular calcium stores, and the inward current induced by OT persists in calcium-free bath solutions (Jiang et al., 2014). In addition, our data shows that OT-induced inhibition of TRPV1 activation was blocked by TTX (**Figure 4**). Therefore, OT inhibition of TRPV1 activity may occur indirectly.

Oxytocin induced an increase of the GABAergic activity in the superficial layer of the spinal dorsal horn, an effect blocked by TTX (**Figure 4**). In a previous study, we reported that OT-induced increase of GABA release was mediated by OT receptors (Jiang et al., 2014). Hanack et al. (2015) noted the GABA_B1_ receptor forms a complex with TRPV1 channel limiting the TRPV1-mediated thermal hyperalgesia. Our data demonstrates that TTX prevents the inhibitory effects of OT on capsaicin-induced TRPV1 activation. Saclofen, a GABA_B_ receptor blocker, prevented the inhibitory effect of OT on capsaicin-induced TRPV1 activation as well (**Figure 4**). In addition, we found that TRPV1 was colocalized with GABA_B_ receptors in superficial layers of rat spinal cords (**Figure 5**). These results suggest that OT inhibits presynaptic TRPV1 activation via presynaptic GABA_B_ receptor activation in the spinal cord.

TRPV1 expression in nociceptors is altered in different models of neuropathy under pathological conditions (Biggs et al., 2007; Kim et al., 2008). TRPV1 protein level up-regulation was significantly suppressed upon OT administration after pSNL surgery (**Figure 7**), suggesting that OT regulates TRPV1 expression levels in the spinal cord under neuropathic pain conditions as well; this may be the mechanism which allowed amelioration of neuropathic pain to continue 2 days after application of OT. Behavioral tests further indicated that OT significantly reduced capsaicin-induced licking behaviors (**Figure 6**), and the antinociceptive effects of OT on thermal hyperalgesia in neuropathic pain became significantly weaker in TRPV1KO mice (**Figure 8**). These results further support the hypothesis that TRPV1 is involved in the analgesic mechanism of OT.

The OT receptor is functionally coupled to the G_q/11_ class GTP binding protein, which leads to the generation of inositol trisphosphate and 1,2-diacylglycerol. Inositol trisphosphate triggers calcium release from intracellular stores, whereas diacylglycerol stimulates protein kinase C (Gimpl and Fahrenholz, 2001). We have previously found that OT depolarized partial neurons via OT receptors, but not vasopressin-V_1A_ receptors, in the superficial layers of the spinal dorsal horn (Jiang et al., 2014). Lin et al. (2017) also found that OT caused membrane depolarization in CA3 pyramidal neurons via OT receptor. In the spinal cord, OT receptor activation results in an alteration in membrane permeabilities to K^+^ and/or Na^+^, which is mediated by phospholipase C and inositol 1,4,5-triphosphate-induced intracellular calcium release (Jiang et al., 2014). This depolarization may result in the enhancement of inhibitory transmission. Regarding the regulation of TRPV1 expression by OT, the mechanism is still unclear. OT has been reported to reduce pMEK1/2 levels (Jurek et al., 2012). The downstream of GABA_B_ receptor is also involved a Ras-Raf-MEK-ERK pathway (Port et al., 2013). Moreover, TRPV1 expression is regulated by MAPK and PI3K (Han et al., 2012). Therefore, it is possible that OT prevents the up-regulation of TRPV1 in the spinal cord through the MAPK pathway; further studies are warranted in this area.

## Conclusion

Our study may suggest that OT relieves neuropathic pain through GABA release and presynaptic TRPV1 inhibition in the spinal cord. Together with the endogenous release of OT following peripheral nerve injury, OT and its receptor system may be an intriguing target for the treatment and prevention of neuropathic pain.

## Author Contributions

CJ and ZC were responsible for the concept and design of the study. WS, QZ, XB, XF, XH, XC, TL, JG, LX, JJ, DX, and YH were involved with experimental and analytical aspects of the manuscript. CJ, ZC, and WS performed the data interpretation, presentation, and writing of the manuscript.

## Conflict of Interest Statement

The authors declare that the research was conducted in the absence of any commercial or financial relationships that could be construed as a potential conflict of interest.
